# DATA-DRIVEN IMPROVEMENTS TO DEMENTIA DISEASE DETECTION STRATEGIES IN AN FQHC SETTING

**DOI:** 10.1093/geroni/igac059.2748

**Published:** 2022-12-20

**Authors:** Theresa M Sivers-Teixeira, Patrick Tabon, Jessie Yuan, Erin Thayer, Sonali Matta, Bonnie Olsen

**Affiliations:** University of Southern California, Alhambra, California, United States; University of Southern California School of Pharmacy, Los Angeles, California, United States; Eisner Health, Los Angeles, California, United States; Keck School of Medicine of University of Southern California, Alhambra, California, United States; Eisner Health, Los Angeles, California, United States; Keck School of Medicine of University of Southern California, Alhambra, California, United States

## Abstract

Despite higher prevalence of dementing illnesses in Latina/o populations, detection is lower compared to white populations, compounding racial/ethnic and economic disparities. Undetected illness compromises effective delivery of primary care and impedes linkages to much needed services. Development of robust detection and culturally sensitive management of cognitive impairment is critical for adequate care of this population. The Geriatric Workforce Enhancement Program at the University of Southern California (USC) partnered with Eisner Health, a Federally Qualified Health Center, serving a primarily immigrant, Spanish-speaking, uneducated, urban older adult population, to help achieve their goal of becoming an Age-Friendly Health System to prioritize identification and management of cognitive impairment. Over 14 months, a sustainable clinic workflow was developed and implemented to detect, evaluate, diagnose, and develop care plans for patients and their care partners. Staff and provider education was delivered through didactics, workshops, case reviews, and at-elbow training. Additional efforts focused on EHR optimization and alignment of existing clinical resources. Frequency data was extracted using i2iTracks software reflecting pre and post-implementation. Results show a higher percentage of patients diagnosed with cognitive impairment in the post-implementation period (7.35%) compared to pre-implementation (4.05%). Detailed data tracking the volume of patients engaged at each step of the workflow supports meaningful analysis of barriers and opportunities for optimization, such as how and where additional resources and efforts will yield the greatest effects. Data-driven strategies such as these, strengthen efficiency and effectiveness of the collaborative process and result in sustainable outcomes for this underserved population.

